# *Plasmodium vivax *trophozoites insensitive to chloroquine

**DOI:** 10.1186/1475-2875-7-94

**Published:** 2008-05-27

**Authors:** Wesley W Sharrock, Rossarin Suwanarusk, Usa Lek-Uthai, Michael D Edstein, Varakorn Kosaisavee, Thomas Travers, Anchalee Jaidee, Kanlaya Sriprawat, Ric N Price, François Nosten, Bruce Russell

**Affiliations:** 1 International Health Program, Infectious Diseases Division, Menzies School of Health Research and Charles Darwin University, Darwin, Australia; 2 Laboratory of Malaria Immunobiology, Singapore Immunology Network, Biopolis, Agency for Science Technology and Research (A*STAR), Singapore; 3 Department of Parasitology, Faculty of Public Health, Mahidol University, Bangkok, Thailand; 4 Department of Therapeutic evaluation, Australian Army Malaria Institute, Brisbane, Australia; 5 Shoklo Malaria Research Unit, Mae Sod, Thailand; 6 Centre for Tropical Medicine, Nuffield Department of Clinical Medicine, University of Oxford, CCVTM, Oxford OX3 7LJ, UK; 7 Faculty of Tropical Medicine, Mahidol University, Rajvithi Road, Bangkok Thailand

## Abstract

**Background:**

*Plasmodium vivax *is a major cause of malaria and is still primarily treated with chloroquine. Chloroquine inhibits the polymerization of haem to inert haemozoin. Free haem monomers are thought to catalyze oxidative damage to the *Plasmodium *spp. trophozoite, the stage when haemoglobin catabolism is maximal. However preliminary *in vitro *observations on *P. vivax *clinical isolates suggest that only ring stages (early trophozoites) are sensitive to chloroquine. In this study, the stage specific action of chloroquine was investigated in synchronous cryopreserved isolates of *P. vivax*.

**Methods:**

The *in vitro *chloroquine sensitivity of paired ring and trophozoite stages from 11 cryopreserved *P. vivax *clinical isolates from Thailand and two *Plasmodium falciparum *clones (chloroquine resistant K1 and chloroquine sensitive FC27) was measured using a modified WHO microtest method and fluorometric SYBR Green I Assay. The time each stage was exposed to chloroquine treatment was controlled by washing the chloroquine off at 20 hours after the beginning of treatment.

**Results:**

*Plasmodium vivax *isolates added to the assay at ring stage had significantly lower median IC_50s _to chloroquine than the same isolates added at trophozoite stage (median IC_50 _12 nM vs 415 nM *p *< 0.01). Although only 36% (4/11) of the SYBR Green I assays for *P. vivax *were successful, both microscopy and SYBR Green I assays indicated that only *P. vivax *trophozoites were able to develop to schizonts at chloroquine concentrations above 100 nM.

**Conclusion:**

Data from this study confirms the diminished sensitivity of *P. vivax *trophozoites to chloroquine, the stage thought to be the target of this drug. These results raise important questions about the pharmacodynamic action of chloroquine, and highlight a fundamental difference in the activity of chloroquine between *P. vivax *and *P. falciparum*.

## Background

*Plasmodium *spp. derive most of their nutritional requirements from the digestion of host erythrocyte haemoglobin. This catabolic process results in the release of toxic free haem. In response to this oxidative threat, *Plasmodium *spp. cross-link free haem monomers to form an inert polymer known as haemozoin or malaria pigment. It is generally thought that the catalytic activity of haem polymerase is the primary target of chloroquine [1,2]. The majority of studies on the mechanism of chloroquine have used *in vitro *cultures of *Plasmodium falciparum *as a model. Despite some controversy regarding the stage specificity of chloroquine [3,4], most agree it is active against the *P. falciparum *trophozoite stage, when haemoglobin catabolism is maximal [5,6]. Unlike *P. falciparum*, it is not yet possible to continuously culture *Plasmodium vixax*, consequently little is known about the mode of action of chloroquine against this species. Despite the emergence of resistance [7], chloroquine is still widely used as the first line of treatment of vivax malaria, due to its relatively good safety profile and low cost. *Ex vivo *studies on clinical *P. vivax *isolates suggest that, in contrast to *P. falciparum*, chloroquine has little effect on the trophozoite stage. Powell and Burgland [8] and recently Suwanarusk *et al *[9] have shown that the chloroquine susceptibility of *P. vivax *depends on the stage of parasite initially exposed to the drug. Isolates that are predominantly at the ring stage (ring to trophozoite ratio RT>1) have a significantly lower IC_50 _than isolates with a RT<1, even if a paired analysis is considered [10]. However these observations on stage specificity are confounded by an unequal time exposure to chloroquine, RT>1 isolates being exposed to chloroquine for ~40 hours before harvest, as opposed to RT<1 isolates which are only exposed for ~24 hours. The objective of this study was to better understand the stage specific action of chloroquine against *P. vivax *using synchronous cryopreserved isolates and a uniform drug exposure time.

## Methods

### *Plasmodium vivax *isolate collection and *P. falciparum *clones

The eleven cryopreserved isolates of *P. vivax *used in this study were obtained from Mae Sod District, Tak Province, located on the North Western border of Thailand. These isolates were collected as part of an earlier published study by Kosaisavee *et al *[11]. All *P. vivax *isolates were obtained and used in accordance with a protocol approved with by the ethical Committee on Human Rights Related to Human Experimentation, Mahidol University, Bangkok. Samples were only taken after written consent was given and the study was explained in Karen, Myanmese or Thai. Isolates were cryopreserved and thawed as described previously [11]. The microscopic speciation of the *P. vivax *isolates were cross-checked using a real-time PCR methodology [12]. Two *P. falciparum *clones, K1 (chloroquine resistant) and FC27 (chloroquine sensitive) were used to quality control the drug plates and also served as a *P. falciparum *comparator for the *P. vivax *assays.

### *In vitro *culture and stage sensitivity assays

A modified WHO schizont maturation assay was used to test the antimalarial susceptibility of *P. vivax *and *P. falciparum *isolates as described previously [13]. A 2% haematocrit Blood Media Mixture (BMM), consisting of McCoy's 5A and 20% AB+ human serum was made for *P. vivax *and *P. falciparum *isolates. 200 μl of BMM, was added to each well of pre-dosed drug plates containing serial dilutions chloroquine (3 to 2,992 nM). Pre-dosed drug plates containing the BMM were placed in a gas chamber containing 5% CO_2_, 5% O_2 _and 90% N_2 _at 37.5°C, until ≥ 50% of parasites in the drug-free control had matured to schizonts (40 hours).

**Figure 1:**
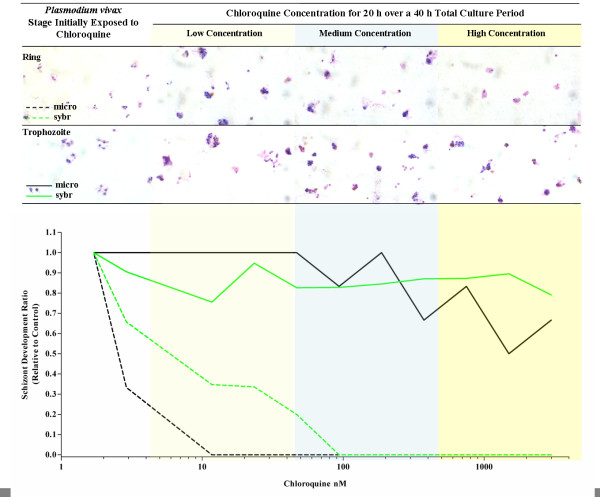
**The inhibition of *Plasmodium vivax *schizont development at increasing concentrations of chloroquine dependent on the initial stage (Ring or Trophozoite) exposed to 20 hours of chloroquine over a 40 hour culture period**. The photomicrographs of thick films, show representative examples of drug effect on *Plasmodium vivax *at low medium and high concentrations of chloroquine. The black (microscopic) and green lines (SYBR Green I) represent the median inhibition of schizont development relative to a drug free control. Medians lines are only derived from assays with paired SYBR Green I and microscopic results.(N = 4).

**Figure 2:**
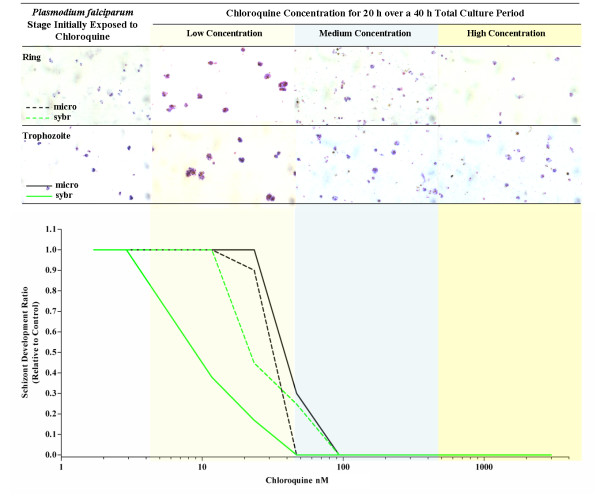
**The inhibition of *Plasmodium falciparum *(Chloroquine resistant K1 clone) schizont development at increasing concentrations of chloroquine dependent on the initial stage (Ring or Trophozoite) exposed to 20 hours of chloroquine over a 40 hour culture period**. The photomicrographs of thick films, show representative examples of drug effect on *Plasmodium falciparum *at low medium and high concentrations of chloroquine. The black (microscopic) and green lines (SYBR Green I) represent the median inhibition of schizont development relative to a drug free control. Medians lines are only derived from assays with paired SYBR Green I and microscopic results.(N = 6).

To assess the stage specificity of chloroquine activity, cryopreserved synchronous *P. vivax *and *P. falciparum *were thawed and split. Half of the sample was added to the pre-dosed drug plates immediately (Figures 1 and 2) and cultured as described above. After 20 hours in the presence of chloroquine, this ring stage treatment was washed once in RPMI 1640 and returned to the incubator with fresh chloroquine free media, for an additional 20 hours. The other half of the thawed isolate was added to a flask and cultured for 20 hours in chloroquine free media. After 20 hours, the predominantly trophozoite isolate, which had been matured in the flask, was added to the pre-dosed plates. Both treatments were harvested approximately 40 hours after the initial incubation. The effect of chloroquine on rings and trophozoites (chloroquine exposure for 40 hours) was tested in *P. falciparum *by not washing the ring stage treatment in an additional set of duplicate wells. Due to a limited amount of cryopreserved *P. vivax *sample available for testing, the effect of chloroquine over the entire 40 hours culture period used the original field *in vitro *CQ sensitivity data collected on the 11 isolates in 2004 [11]. All *P. falciparum *clone experiments were conducted in triplicate, quadruplicate or sextuplicate.

### Assessing schizont maturation

Schizont maturation was determined by two methods: using microscopy [13,14] and a modified SYBR Green I based assay [15]. Microscopic evaluations were based on earlier work by Rieckmann *et al *[14] and were done by counting the number of mature schizonts with ≥five more nuclei out of a total 200 asexual parasite in every thick film. The percentage schizont counts read in the drug wells were then expressed as a proportion of that of drug free controls. The SYBR Green I assay used was modified from the method previously described by Smilkstien *et al *[15] After the 40 hours incubation period, supernatant was removed and replaced with unsupplemented RPMI 1640 (Gibco/Invitrogen), and a 50 μL of a fluorochrome/lysis mixture (consisting of 50 mM TRIS-base, 12.5 mM EDTA, 0.02% w/v saponin (Sigma Chemical Co., St Louis, MO, USA), 0.2% v/v Triton-X100, and a 1:2000 dilution of freshly thawed stock (×10 000) SYBR Green I (Invitrogen). This was dark incubated at room temperature for at least 60 minutes, and read at wavelengths of 485 nm and 535 nm excitation and emission respectively. The relative fluorescence units (RFU) output from each well had time zero fluorescence removed, and was normalized to the RFU from drug free controls.

### Chloroquine concentrations

The amount of chloroquine added to the plates and the effectiveness of the chloroquine wash step were investigated by high performance liquid chromatographic (HPLC) analysis [16]. Two chloroquine plates were incubated with 200 μl of 2% haematocrit BMM for 20 hours. One plate was subjected to a wash as defined above, and the other plate was not. Complete RPMI medium was then added to both plates were and the contents from triplicate wells were combined and transferred to 2 ml cryovials. The vials were stored at -80°C and shipped on dry-ice to the Australian Army Malaria Institute where they were stored at -80°C until analysis. Chloroquine concentrations were measurement by normal-phased HPLC using fluorescence detection. The lower limit of quantification was 5 ng/ml, using 0.5 ml samples.

### Analysis

The growth responses for each of the treatments and assays were converted to a percentage of the drug free positive control 40 hours. The background 0 hours was subtracted from each of the data points. IC_50 _data was calculated using WinNonLin (version 4.1, Pharsight) using a compiled Pharmacodynamic, Inhibitory Effect Sigmoid E_max _Model. IC_50 _data was only used from curves where the predicted Emax = 1 +/- 0.3 and the E_0 _= 0 +/- 0.3. Non parametric tests; Wilcoxon (2 related samples) or Friedman's (3 related samples) were used to compare the median IC_50 _data (SPSS ver.14.0).

## Results

**Figure 3:**
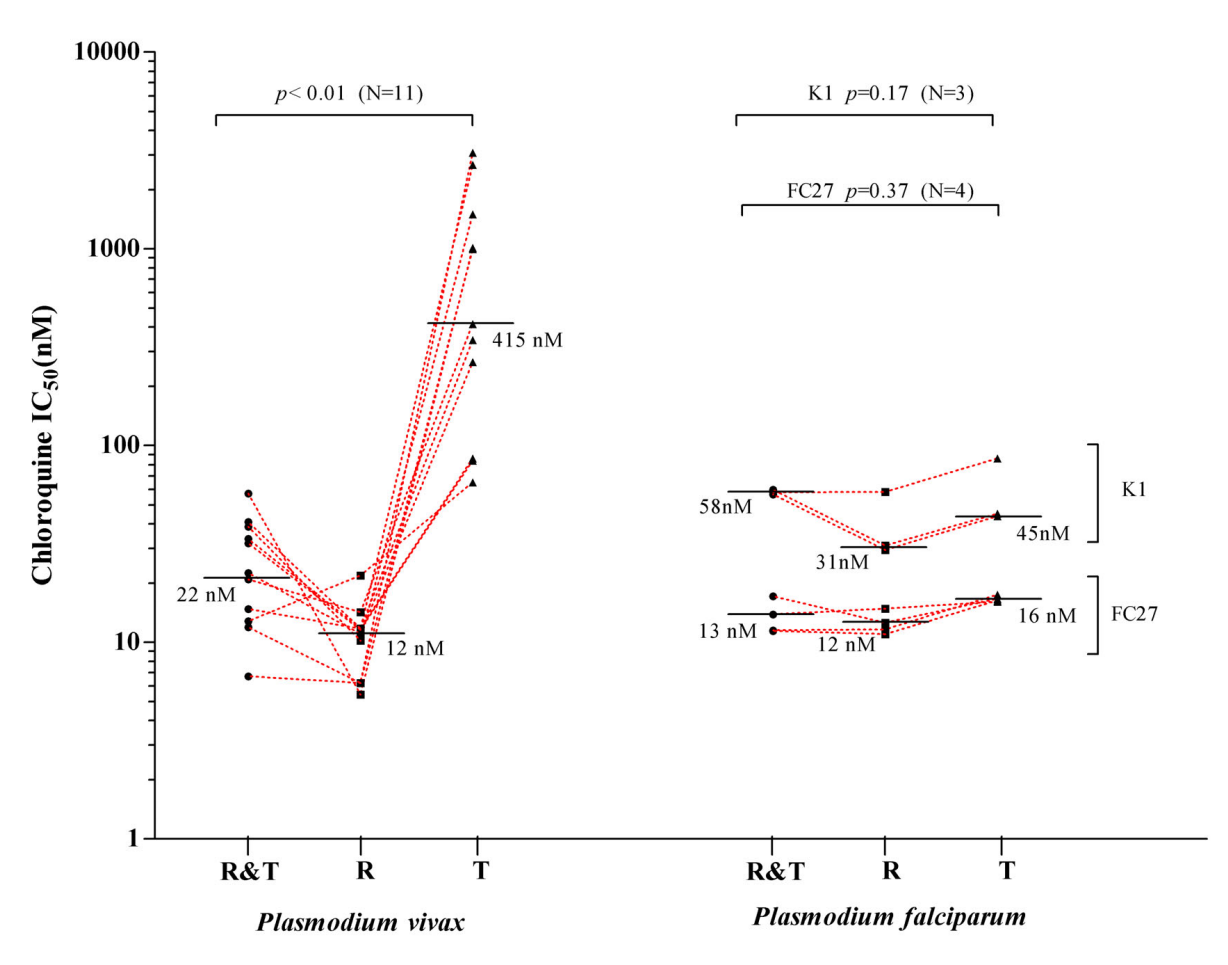
**The sensitivity of *Plasmodium vivax *isolates and *Plasmodium falciparum *clones (K1 and FC27) to chloroquine (CQIC^50^nM) when**; rings and trophozoites are exposed to chloroquine for 40 hours (R&T); exposed to chloroquine for 20 hours at the ring stage (R) and trophozoite stage (T). Red dotted lines connect related samples. Solid black lines represent the median CQIC^50^nM for each treatment.

At least 50% of *Plasmodium vivax *trophozoites developed to mature schizonts in spite of 20 hours exposure to 2,992 nM of chloroquine during the trophozoite stage of the life cycle (Figure 1). In contrast, *P. vivax *ring stage and *P. falciparum *ring and trophozoite stage treatments were all sensitive to chloroquine concentrations as low as 100 nM (Figures 1 and 2). All of the *P. falciparum *SYBR Green I assay were successful (6/6) and the derived IC_50_s were similar to the microscopic method. Although only 36% (4/11) of the *P. vivax *SYBR Green I assays were successful (insufficient signal to background ratio) and these data showed similar trends to the microscopic assay (Figure 1). Due to the low success rate of the *P. vivax *SYBR Green I assay, further analysis in this study is limited to the microscopic assay. The *Plasmodium vivax *isolates added to the assay at ring stage irrespective of the length of chloroquine exposure had significantly lower median IC_50s _to chloroquine than the same isolates added at trophozoite stage (median IC_50 _12 nM vs 415 nM p < 0.01) (Figure 3). The sensitivity of *P. falciparum *(K1 and FC27) to chloroquine was not significantly effected by the stage first exposed or time of chloroquine exposure (median IC_50s _for K1: 31 vs 45 nM p = 0.17 and FC27; 12 vs 16 nM p = 0.37) (Figure 3). The chloroquine IC_50 _values for our K1 clone of *P. falciparum *were considerably less that those published by Elueze *et al *(K1 CQ IC_50_; 214 nM) and Fivelman *et al. *(K1 CQ IC_50 _; 266 nM) [17,18]. It is conceivable that these differences are due to the methodology (microscopy vs hypoxanthine incorporation) and non-linear analysis program used. Kosaisavee *et al. *showed that the mean CQ IC_50 _of K1 determined by the isotopic method was 2.5 fold higher than the microscopic method [11]. For the past five years consistent K1 CQ IC_50_s of less than 150 nM [9] have been recorded on a K1 clone from the Australian Army Malaria Institute, Brisbane, confirmed to be a *pfcrt *CVIET mutant.

HPLC analysis showed that washing of the BMM was successful in reducing the chloroquine concentrations by at least 93%(mean 95.4 ± 2.2%, n = 5 paired samples).

## Discussion

### Chloroquine resistance mechanism in *P. vivax *different to *P. falciparum*

The rapid spread of chloroquine resistant vivax malaria has provided impetus for studies on molecular markers associated with this phenotype [7]. In *P. falciparum *SNPs in *pfcrt *are strongly associated with reduced chloroquine sensitivity [19,20]. However, molecular changes in its *P. vivax *homolog, *pvcg10 *are not linked to changes in the chloroquine sensitivity phenotype of this species [9]. This finding implies that chloroquine resistant *P. vivax *has a different molecular mechanism for avoiding the antimalarial effects of chloroquine. It could be that chloroquine resistant *P. vivax *has a yet to be identified transporter for limiting the effect of chloroquine on haem polymerase, or that that chloroquine has a completely different molecular target not related to the inhibition of haemozoin formation. Data from this and earlier studies support the latter view, by confirming the almost complete absence of chloroquine activity against *P. vivax *trophozoites, the stage where most of the haem polymerase activity occurs.

### Innate resistance of *P. vivax *trophozoites

The stage specific effect of chloroquine shown in this study of chloroquine sensitive *P. vivax *isolates from Thailand, and earlier studies using resistant *P. vivax *isolates from Papua [9,10] demonstrates that this is an innate trait of *P. vivax *that is not restricted to chloroquine resistant strains. Two plausible explanations for the innate resistance of *P. vivax *trophozoites to chloroquine are; firstly an inefficient haem polymerase binding site for chloroquine; or secondly a wild type vacuolar transporter system that reduces the intra-vacuolar concentration of chloroquine below the threshold for haem polymerase inhibition. Interestingly, innate resistance to another antimalarial, sulphadoxine has already been described in *P. vivax*. Innate resistance to the antifolate sulphadoxine is due to the change of one residue at v585 on the *pvdhps *sulphadoxine binding site (relative to A613 *pfdhps*) conferring a significantly lower sensitivity to sulphadoxine than *P. falciparum*. Any polymorphisms in *pvdhps *will only modulate the level of this resistance [21,22]. However, the ring stage and trophozoites of *P. vivax *are equally insensitive to sulphadoxine, unlike the stage specific effect of chloroquine on the ring stages of *P. vivax*.

### Chloroquine sensitive ring stage of *P. vivax*: possible molecular targets

Aside from variation in the chloroquine sensitivity phenotype between *P. vivax *strains and universal trophozoite resistance, chloroquine effects the ring stage of *P. vivax*. Therefore, the most important question raised by this study is, by which mechanism does chloroquine effect ring stage *P. vivax*? Although most research on chloroquine action has focused on the digestive vacuole, earlier studies suggest that the primary antimalarial action of chloroquine is within nucleus [23-27]. Of note, Picot *et al *showed that after CQ treatment, oligonucleosomal DNA fragmentation was observed with a chloroquine sensitive strain of *P. falciparum*, suggesting CQ action on the nucleus leading to apoptosis [28]. CQ also interferes with the DNA synthesis step of the repair process, most likely due to direct binding to repair substrates [29]. Recent studies have shown that chloroquine can destabilize the mRNA in eukaryotic cells by a pH-dependent mechanism [30]. As all of the above processes are vital to the early stages of intra-erythrocytic life, it is hypothesized that the ring stage of *P. vivax *is vulnerable to the effect of chloroquine on its nucleus as compared to the trophozoite stage when most of the cellular infrastructure is already established with a transporter system capable of limiting further chloroquine damage. If this is the case, intra species variations in *P. vivax *chloroquine sensitivity might be associated with differential rates of parasite development, faster developing parasites capable of rapidly expressing the protein systems which limit the effects chloroquine on its nucleus. Indeed recent findings indicate that rapidly growing *P. vivax *[9] and *P. falciparum *[31] are less sensitive to chloroquine.

## Conclusion

The diminished activity of chloroquine on *P. vivax *trophozoites, the stage thought to be the central target of this drug, raises important questions about the pharmacodynamic action of chloroquine. The established biological and genetic differences between *P. vivax *and *P. falciparum *are further highlighted by the results of this study. Clearly future studies are needed to determine the specific mechanism of chloroquine activity in *P. vivax*. However, the application of models purely focusing on the digestive vacuole is unlikely to succeed in *P. vivax*.

## Authors' contributions

WSS developed, helped in designing this study, optimized and conducted the experiments, and drafted the manuscript, UL, VK, KS and RS processed and cryopreserved the isolates, read slides, conducted the PCR methods, UL also obtained the ethical clearances for this study, FN coordinated the field studies, sample collection, clinical support and provided intellectual guidance though out this study, ME and TT conducted the HPLC experiments, BR and RNP conceived the study, designed and participated in the experiments and the drafting of the manuscript. All authors read and approved the final manuscript.
